# Low Nitrogen Application Enhances Starch-Metabolizing Enzyme Activity and Improves Accumulation and Translocation of Non-structural Carbohydrates in Rice Stems

**DOI:** 10.3389/fpls.2018.01128

**Published:** 2018-07-31

**Authors:** Guohui Li, Qiuqian Hu, Yange Shi, Kehui Cui, Lixiao Nie, Jianliang Huang, Shaobing Peng

**Affiliations:** ^1^National Key Laboratory of Crop Genetic Improvement, Ministry of Agriculture Key Laboratory of Crop Ecophysiology and Farming System in the Middle Reaches of the Yangtze River, College of Plant Science and Technology, Huazhong Agricultural University, Wuhan, China; ^2^Hubei Collaborative Innovation Center for Grain Industry, Jinzhou, China

**Keywords:** nitrogen application, starch-metabolizing enzymes, non-structural carbohydrates, grain filling, rice (*Oryza sativa* L.)

## Abstract

More than 4 billion inhabitants in Asia depend on rice for 35–60% of the calories consumed in their diets, but new rice cultivars frequently do not reach expected yields because of poor rice grain filling. Here, we quantified the activities of enzymes involved in starch metabolization in rice to investigate the mechanisms regulating the accumulation and translocation of stem non-structural carbohydrates (NSC) under different levels of nitrogen fertilizer application. A pot experiment was conducted using two rice cultivars, Liangyoupeijiu (LYPJ) and Shanyou63 (SY63), under high and low nitrogen applications. Compared with high nitrogen application (HN), low nitrogen application (LN) increased stem NSC concentration before the heading stage and NSC translocation during the grain filling stage; concomitantly, LN significantly shortened the active grain filling period and increased the grain filling rate in superior spikelets. Compared with the LYPJ cultivar, SY63 exhibited a higher grain weight, higher grain filling percentage, and higher stem NSC concentration before heading and greater NSC translocation after heading. During the period between panicle initiation and heading, the activities of adenosine diphosphate-glucose pyrophosphorylase (AGP), starch synthase (StS), and starch branching enzyme (SBE), all enzymes involved in starch synthesis, increased under the LN treatment and positively correlated with increases in stem NSC. During grain filling, the activities of enzymes involved in starch-to-sucrose conversion [α-amylase, β-amylase, and sucrose phosphate synthase (SPS)] increased under the LN treatment and positively correlated with stem NSC remobilization. Overall, the investigated enzymes exhibited higher activities in SY63 than in LYPJ. Our results suggest that low nitrogen increases the activities of AGP, StS, SBE, α-amylase, β-amylase, and SPS, leading to increased accumulation and remobilization of stem starch and NSC in SY63. We conclude that calculated reductions in nitrogen application and the choice of an appropriate cultivar may improve rice grain yields via enhanced stem NSC accumulation and translocation, thereby reducing the costs and increasing the sustainability of rice production.

## Introduction

Rice (*Oryza sativa* L.) provides 35–60% of the calories consumed by the more than 4 billion inhabitants of Asia (Fageria, 2007; Yorobe et al., 2016). Growing populations, climate change (Nelson et al., 2016), and limits on the availability of arable land (Beddington, 2010) make it increasingly difficult to meet food demands and under the changing climate currently (Nelson et al., 2016). It’s not viable to improve rice yield through the expansion of arable land in the future; future efforts to meet rising food demands must therefore rely on maximizing rice yields.

The emergence of new rice varieties through breeding has raised rice yield potential to unprecedented levels. High-yield varieties include those developed by the International Rice Research Institute, such as hybrid rice and “super” rice (Cheng et al., 2007; Peng et al., 2008). These cultivars, however, frequently do not reach expected yields because of poor rice grain filling. Grain filling in rice depends on carbon assimilation in photosynthetic leaves and the remobilization of NSC temporarily stored in leaf sheaths and culms prior to heading. NSC stored in stems contributes an estimated 1–28% to rice yields (Pan et al., 2011). Improvements in the accumulation of NSC in stems and their translocation to rice grains increases sink capacity, grain filling rates, and grain yields (Yang and Zhang, 2006; Fu et al., 2011). Therefore, it is important to elucidate the mechanisms of stem NSC accumulation and translocation to maximize rice yield potentials and increase future rice yields.

Environmental factors influence the accumulation and translocation of stem NSC in rice. NSC translocation from stems increased during periods of environmental stress, such as high or low solar radiation (Okawa et al., 2003), heat stress (Morita and Nakano, 2011), and water deficiency (Yang et al., 2001a), when conditions caused photosynthesis to decrease. Nitrogen (N) plays a role in many physiological processes in crops, including photosynthesis and assimilate allocation, plant growth and development, and grain yield (Mae, 1997). The accumulation and translocation of stem NSC has been closely associated with the rate and timing of nitrogen applications, and low nitrogen conditions have increased stem NSC accumulation and translocation (Fu et al., 2011; Pan et al., 2011). Excessive nitrogen applications are not conducive to NSC accumulation and translocation and do not result in maximum yields (He et al., 2001). Although the activities of starch metabolizing enzymes have been shown to be influenced by the amount of nitrogen fertilizer applied to crops, the activity of SBE, which is important for starch accumulation during the heading period, decreased under high-nitrogen conditions (Hirano et al., 2005). Moreover, N-starvation induced the expression of genes encoding starch synthesis enzymes (Dian et al., 2003). These reports suggest that N supply affects starch synthesis by altering the expression and activity of the starch metabolizing enzymes involved in NSC accumulation and translocation. However, we do not yet have a full understanding of the mechanisms underlying these effects.

N fertilizers are often applied to achieve high grain yields, but excess N can also produce negative consequences, such as low nitrogen-use efficiency, environmental pollution and water eutrophication, diminishing economic returns on the amount of fertilizers applied, poor quality of the rice grains produced, and reduced grain yields due to pest damage (Peng et al., 2002). Studies have shown that appropriate reductions in nitrogen fertilizer applications may still maintain the balance between grain yields and nitrogen-use efficiency (Peng et al., 2002; Ju et al., 2009; Ju and Christie, 2011). It is possible to reduce environmental risks by improving N management in intensive Chinese agricultural systems (Ju et al., 2009). Zhang (2007) proposed the development of green super rice, which produces high yields even with low inputs of water, fertilizers, and pesticides, promising to reduce the consumption of chemical fertilizers. Sustainable agricultural systems demand reduced N application and can be achieved by developing high yield crops and management practices that are less dependent on heavy fertilizer application.

Starch, the main storage form of NSC in the parenchyma cells of leaf sheaths and culms, accumulates during the vegetative growth stage of rice plants and decreases sharply during grain filling (Ishimaru et al., 2007). During starch accumulation in rice stems before heading, the enzymes AGP (Enzyme Commission number 2.7.7.27), StS (EC 2.4.1.21), and SBE (EC 2.4.1.18) play key roles in the regulation of starch synthesis (Nakamura et al., 1989; Keeling and Myers, 2010). Starch levels in leaf sheaths and culms have been observed to parallel StS activity (Perez et al., 1971) and starch accumulation in leaf sheaths correlate with AGP and SBE activities and mRNA levels (Watanabe et al., 1997; Hirose et al., 1999).

After heading, stem starch is the first NSC source to be converted to sucrose, the primary sugar transported from source to sink in most plants (Braun et al., 2014). Starch is primarily hydrolyzed by α-amylase (EC 3.2.1.1) and β-amylase (EC 3.2.1.2) (Ishimaru et al., 2004). During the post-anthesis stage, increased gene expression and activity of α-amylase contributes to rapid starch degradation and increased remobilization from rice stems (Sugimura et al., 2015). SPS (EC 2.4.1.14) and sucrose synthase (SS; EC 2.4.1.13) are the primary enzymes involved in sucrose synthesis (Wardlaw and Willenbrink, 1994; Lunn and MacRae, 2003), while SS and invertase (acid and neutral invertase; EC 3.2.1.26) are the primary enzymes involved in sucrose degradation (Lowell et al., 1989; Li et al., 2017). The enzymes involved in sucrose synthesis and degradation regulate the sucrose content of stems and NSC translocation from stems to developing grains post-anthesis.

Different rice cultivars display large variations in their capacities for NSC accumulation in, and translocation from, stems. Fujita and Yoshida (1984) reported that NSC levels in leaf sheaths and culms of tall and short rice varieties were significantly different. Genotypes with high grain weights also exhibit a higher level of stem NSC translocation to the panicle than genotypes with lower grain weights (Samonte et al., 2001). The contribution of stem NSC to final grain yield is an estimated 10–40% in rice (Yang and Zhang, 2010a). Among recombinant inbred rice lines from Zhenshan 97 and Minghui 63, the contribution of NSC to grain yield ranged from 1 to 28% under low nitrogen and 1–15% under normal nitrogen treatments (Pan et al., 2011). These reports demonstrate that stem NSC translocation and contribution to grain yield depends on genotype. However, the underlying mechanisms for these genotypic differences remain unknown.

Rice plants often accumulate NSC before anthesis, which contributes to grain filling post-anthesis via NSC translocation (Pan et al., 2011). NSC translocation from stems to grains under low N application levels may partially meet plant demands for assimilates during grain development (Pan et al., 2011; Ju et al., 2015). However, we need an improved understanding of the physiological effects of LN on the regulation of stem NSC accumulation and translocation. In the current study, we conducted pot experiments using two rice cultivars to identify the activities of enzymes involved in starch – sucrose conversion, which regulate stem NSC accumulation and translocation in rice under different nitrogen application levels.

## Materials and Methods

### Plant Materials and Growth Conditions

Two rice cultivars, Liangyoupeijiu (LYPJ, *Oryza sativa* L. ssp. *indica*) and Shanyou63 (SY63, *Oryza sativa* L. ssp. *indica*), were used in a pot experiment at Huazhong Agricultural University, Wuhan, China. LYPJ and SY63 were two elite rice hybrids that were widely planted in China (Lv et al., 2017), with significant genetic variations in grain filling percentage and grain weight (Yang and Zhang, 2010b). Compared with LYPJ (two-line hybrid), SY63 (three-line hybrid) had higher grain filling percentages and grain weights of whole panicle (8.6 and 8.7%), superior spikelets (4.4 and 1.7%), and inferior spikelets (14.4 and 18.5%), respectively (Yang and Zhang, 2010b).

Germinated seeds were seeded to nursery plates containing sandy loam soil on 23 May 2015. On 17 June 2015, three uniform, 25-day-old seedlings were transplanted into each of 72, 12.0 L pots (diameter 24.5 cm; height 25.5 cm) filled with 10.0 kg soil. The soil used was a clay type soil and had the following properties: pH 7.1, 7.0 g organic matter kg^-1^, 0.07 g total N kg^-1^, 6.3 mg Olsen P kg^-1^, and 127.0 mg exchangeable K kg^-1^. Considering that the main objective of the study was to investigate the underlying mechanism for regulation of N application rates on stem carbohydrates accumulation and translocation, the two N treatments were used, between which was 10 times differences, with the purpose of making significant differences in physiological traits in response to N application rates. Namely, low N and high N application rates were 0.26 g N pot^-1^ (LN) and 2.6 g N pot^-1^ (HN) in the form of urea, which were equal to N application rates of 21 kg ha^-1^ and 210 kg ha^-1^ in farmer field, respectively. Each N treatment and each cultivar was repeated three times. The nitrogen fertilizer applications were split-applied at a ratio of 4:3:3 at three different times: basal (1 day before transplanting), tillering stage (approximately 36 days after germination), and at panicle initiation (approximately 66 days after germination). Phosphorous (P) and potassium (K) in the form of KH_2_PO_4_ were applied as basal fertilizers at rates of 1.50 and 1.89 g pot^-1^, respectively. Basal fertilizers were applied by thorough mixing into the soil before transplanting; subsequent fertilizers were applied in a top-dressed manner by dissolving them in water and then irrigating. Eighteen pots were used for each of the four treatments testing rice variety and N level, for a total of 72 pots. During the entire experiment, plants received well water, maintaining a water depth of at least 2 cm. Pests and diseases were controlled using chemical pesticides.

### Sampling for Dynamic Changes in Enzyme Activity and Sugar Content in Stems

Upon panicle initiation (PI, approximately 66 days after germination) when the average length of young panicles was about 1 mm (as determined by checking several young panicles under a magnifier), several main and large stems were chosen and tagged for each treatment. Panicles from main or large stems that flowered on the same day were chosen and tagged on the heading date (HD, approximately 96 days after germination). Six tagged main or large stems (culms and sheaths) from six plants in each treatment were sampled at 5-day or 6-day intervals from panicle initiation to maturity. Considering the effect of plant number on available N for each plant and experiment scale, only two large stems were tagged from each plant from the same pot for sampling, one for each time point. After the two samplings, the plants were not used for any other sampling.

Three sampled stems were immediately frozen in liquid nitrogen and then stored at -80°C for determination of enzyme activity. The other stems were oven-dried at 80°C to constant weight and ground for the measurement of soluble sugars, starch, and sucrose. In total, we collected six samplings for measuring enzyme activity during the PI stage (day of PI, and 5, 10, 15, 20, and 26 days after PI) and six samplings for measuring enzyme activity between heading and maturity (day of heading, and 6, 12, 18, 24, 30 days after heading).

### Sampling for Grain Filling Rate and Grain Yield

During the spikelet ontogeny, superior spikelets are often referred to ones located on apical primary branches, and flower first and fill fast; inferior spikelets located on proximal secondary branches often have hysteretic nature of grain filling and fill slowly (Fu et al., 2016). After heading, three tagged panicles were sampled at 4-day intervals until maturity, and then superior and inferior spikelets were separately collected for determination of grain filling, respectively. All superior and inferior spikelets were oven-dried at 80°C to a constant weight and weighed. The active grain filling period (number of days) was defined as the period of time that grain weight was between 5% (t1) and 95% (t2) of final grain weight. The average grain filling rate (mg grain^-1^ d^-1^) was calculated between t1 and t2 (Zhang et al., 2012).

At both heading and maturity, three entire plants per treatment were sampled and separated into leaves, stems (culms and sheaths), and panicles. All spikelets of the panicles were hand-threshed at maturity. Filled grains and unfilled grains were divided by submersing them in tap water. Empty grains were separated from unfilled grains using a seed winnowing cleanliness instrument. The dry weights of all parts, including leaves, stems, rachises, and spikelets were weighed after being oven-dried at 80°C to a constant weight. The numbers of filled, partially filled, and empty grains were counted, and the 1000-grain weight (g) and percentages of half-filled and fully-filled grains (%) were calculated. Biomass (g plant^-1^) was the total dry weight of the aboveground plant parts, grain yield (g plant^-1^) was the weight of full-filled grains, and harvest index (%) was calculated as the ratio of the grain yield to the biomass.

To determine the contribution of stem NSC to grain yield, stems were ground and measured for contents of soluble sugars, starch, and sucrose.

### Assays for Soluble Sugars, Starch, and Sucrose in Stems

Soluble sugars and starch were determined according to the method described by Li et al. (2017). Briefly, oven-dried plant samples were ground to powder and approximately 100 mg of powder sample was extracted with 5 mL of 80% (v/v) ethanol at 80°C for 30 min; this was repeated three times. After centrifugation, all supernatant was collected in a 100 mL volumetric flask and brought to a final volume of 100 mL by adding distilled water. This solution was used for determination of soluble sugars and sucrose concentrations.

To determine starch concentration, the solid residue in the tube was resuspended in 2 mL of distilled water and placed in a boiling water bath for 15 min. After cooling, 2 mL of 9.2 mol L^-1^ HClO_4_ was added and the tube was stirred occasionally for 15 min. After centrifugation, the supernatant was collected in a 100 mL volumetric flask. These extraction steps were then repeated by adding 2 mL of 4.6 mol L^-1^ HClO_4_ for 15 min. The supernatants were combined and brought to 100 mL final volume with distilled water.

Concentrations of soluble sugars and starch were determined by a colorimetric method using anthrone reagent at 620 nm and a microplate reader (Nano Quant, infinite M200, Tecan, Switzerland). After comparison to a standard curve of glucose, concentrations of soluble sugars and starch were calculated as mg glucose g^-1^ dry weight. Glucose released in the extraction was estimated with anthrone reagent and converted to a starch value by multiplying by 0.9 (Li et al., 2017).

Sucrose was determined according to the method of Wang et al. (2015). The extract was decolorized with activated carbon and purified by filtering. Two-hundred μL of 2 mol L^-1^ NaOH solution was added to 0.4 mL of filtered solution, then the mixture was boiled at 100°C for 5 min. After cooling, 2.8 mL of 9 mol L^-1^ HCl and 0.8 mL of 0.1% resorcinol were added and the reaction mixture was incubated at 80°C for 10 min, followed by determination of absorbance at 480 nm. After comparison to a standard curve of sucrose, the sucrose concentration was calculated as mg g^-1^ dry weight.

The NSC concentration (mg g^-1^ dry weight) of a given plant part is the sum of concentrations of soluble sugars and starch. The total mass of NSC stored in stems (TM; g plant^-1^) was calculated as stem biomass multiplied by NSC concentration. The apparent transferred mass of NSC from stems to grains during grain filling (ATM; g plant^-1^) is the TM at heading minus TM at maturity. The apparent ratio of transferred NSC from stems to grains (AR; %) is the ratio of ATM to TM at heading. The apparent contribution of transferred NSC to grain yield (AC; %) is the ratio of ATM to grain yield. In this article, NSC translocation was described by ATM, AR, AC, and δC_NSC_ (defined as the differences in NSC concentrations at heading and at maturity).

### Enzyme Extraction and Activity Assays for Enzymes Involved in Stem NSC Accumulation During Panicle Initiation

Between the panicle initiation and heading stages, the three tagged stems were sampled every 5 days to measure the activities of three key enzymes involved in starch synthesis (AGP, StS, and SBE). The sampled stems were ground and extracted with a medium composed of 100 mM Hepes-NaOH (pH 7.6), 8 mM MgCl_2_, 5 mM dithiothreitol (DTT), 2 mM ethylene diamine tetraacetic aicd (EDTA), 12.5% (v/v) glycerol, and 5% (w/v) polyvinylpolypyrrolidone (PVPP). The homogenate was centrifuged at 4°C and 12 000 rpm for 20 min and the supernatant was used in an enzyme extraction to determine the activities of AGP, StS, and SBE.

The activity of AGP was determined according to Nakamura et al. (1989). Briefly, 20 μL of enzyme extract was added to 110 μL of reaction medium in an Eppendorf tube containing 100 mM Hepes-NaOH (pH 7.4), 1.2 mM ADP-glucose, 3 mM PPi, 5 mM MgCl_2_, and 4 mM DTT. After incubating at 30°C for 15 min, the reaction was terminated by placing the tube into boiling water for 30 s. The resulting solution was centrifuged at 12,000 rpm for 10 min. Then, 100 μL of the supernatant was mixed with 3 μL of 10 mM nicotinamide adenine dinucleotide phosphate (NADP). The absorbance at 340 nm was measured after addition of phosphoglucomutase (0.4 unit) and glucose-6-P dehydrogenase (0.35 unit). The protein content of the enzyme extract was measured according to Bradford (1976), using bovine serum albumin as the standard. The activity of AGP is expressed as μmol NADPH mg^-1^ protein h^-1^.

Starch synthase activity was measured according to Nakamura et al. (1989). Twenty μL of the enzyme extract was added to 36 μL of reaction medium containing 50 mM Hepes-NaOH (pH7.4), 1.6 mM ADP-glucose, 0.7 mg amylopectin, and 15 mM DTT. After incubating for 15 min at 30°C, the reaction was terminated by placing the mixture in a boiling water bath for 30 s. Then, 20 μL of the mixture was added to 50 mM Hepes-NaOH (pH7.4), 4 mM phosphoenolpyruvate (PEP), 200 mM KCl, 10 mM MgCl_2_, and pyruvate kinase (1.2 unit). After incubating at 30°C for 15 min, the solution was heated in boiling water for 30 s to terminate the reaction and centrifuged at 4°C and 12 000 rpm. Next, 60 μL of supernatant was mixed with 43 μL of 50 mM Hepes-NaOH (pH7.4), 10 mM glucose, 20 mM MgCl_2_, and 2 mM NADP in a tube and incubated for 10 min at 30°C. The absorbance was measured at 340 nm after the addition of hexokinase (1.4 unit) and glucose-6-P dehydrogenase (0.35 unit). The activity of StS is expressed as μmol NADPH mg^-1^ protein h^-1^.

Activity of SBE was determined according to Zhao et al. (2007). The assay was conducted by adding 20 μL of the enzyme extract to 250 μL of extraction medium and 20 μL 0.75% soluble starch. After incubating for 15 min at 37°C, the reaction was terminated by placing the mixture in boiling water for 30 s. The solution was then mixed with 0.4 mL 0.2% HCl and 150 μL of 0.1% I_2_-1% KI after cooling. The absorbance was measured at 610 nm and the remaining starch content was determined by comparing it to the starch standard. The activity of SBE is expressed as mg starch mg^-1^ protein h^-1^.

### Enzyme Extraction and Activity Assays for Enzymes Involved in Stem NSC Redistribution During the Grain Filling Stage

Between the heading and maturity stages, we sampled stems every 6 days to determine the activities of key enzymes involved in starch-to-sucrose conversion (α-amylase, β-amylase, SPS, SS, AI, and NI).

For extraction of α-amylase and β-amylase, frozen stems were ground in pre-cooled 100 mM phosphate buffer (pH 6.5) and centrifuged at 4°C and 12,000 rpm for 20 min. The enzyme activities were determined as described by Bhatia and Singh (2002). Briefly, the assay mixture for α-amylase consisted of 0.6 mL 12.7 mM calcium acetate (pH 6.0), 0.3 mL 0.067% starch solution, and 0.1 mL enzyme extract. The assay mixture was then heated at 70°C for 20 min to inactivate β-amylase. After incubating at 37°C for 20 min, the intensity of color developed with 0.1% I_2_-1% KI was measured at 610 nm. A control sample was prepared using deactivated enzyme boiled for 30 s. For the assay of β-amylase, the assay mixture consisted of 200 mM sodium acetate containing 0.1 mM EDTA (pH 3.6), 0.3 mL 0.067% starch solution, and 0.1 mL enzyme extract. The procedure was the same as for the α-amylase assay. The activities of α-amylase and β-amylase were expressed as mg starch hydrolyzed mg^-1^ protein h^-1^.

Extraction and assay of SPS and SSs were performed according to Rufty et al. (1983) and Wardlaw and Willenbrink (1994), respectively. Briefly, frozen stems were ground in a medium containing 50 mM Hepes-NaOH (pH 7.5), 10 mM MgCl_2_, 1 mM EDTA, 5 mM DTT, 1 mM phenazine methosulfate (PMSF), 1 mM benzamidine, and 3% (w/v) PVPP. After centrifuging at 4°C and 12,000 rpm for 20 min, the supernatant was used for the assay of SPS and SSs. For SPS, 30 μL enzyme preparation was added to a tube of 70 μL reaction medium containing 50 mM Hepes-NaOH (pH 7.5), 3.5 mm UDP-glucose, 3.5 mM fructose-6-P, and 10 mM MgCl_2_. The mixture was then incubated at 30°C and the reaction was terminated after 15 min by adding 100 μL 1.0 mol L^-1^ NaOH. Unreacted fructose-6-P was destroyed by placing the tubes in boiling water for 10 min. After cooling, 0.75 mL of 9 mol L^-1^ HCl and 0.25 mL of 0.1% (w/v) resorcinol were added and the tube was incubated at 80°C for 10 min. Absorbance was measured at 480 nm after cooling. For SSs, the reaction mixture contained 30 μL of enzyme preparation, 30 μL of 100 mM fructose, and 70 μL of medium containing 100 mM Hepes-NaOH (pH 8.5), 5 mM KCl, 5 mM NaCl, and 8 mM UDP-glucose. The reaction mixture was then incubated at 30°C for 15 min. The remaining steps were the same as for SPS determination. The enzyme activity of SPS and SSs were expressed as μmol sucrose synthesized mg^-1^ protein h^-1^.

The assays for AI, NI, and sucrose synthase activity in the cleavage direction (SSc) followed the protocol described by Li et al. (2017). Enzyme activity was expressed in μmol glucose produced mg^-1^ protein h^-1^.

### Statistical Analysis

The mean values of investigated traits described here were compared based on the least significant difference (LSD) test at a 5% probability level using the Statistix 9 software package (Analytical software, Tallahassee, FL, United States). Linear correlation analysis was used to evaluate the relationships of enzyme activities with stem NSC accumulation and translocation using the SigmaPlot 10.0 software package (SPSS Inc., Chicago, IL, United States). To determine the relations of enzyme activities with NSC accumulation during the rapid accumulation of stem starch and NSC 0–20 days after panicle initiation, we correlated enzyme activities with starch/NSC accumulation using the differences between starch/NSC concentrations on the date of panicle initiation and days 5, 10, 15, and 20 afterward (*n* = 4 for each cultivar, *n* = 8 for each nitrogen treatment). To determine the relations of enzyme activities with NSC translocation during the rapid decrease in stem starch and NSC 6–24 days after heading, we correlated enzyme activities involved in starch/NSC decreases using the differences between starch/NSC concentration at heading and days 6, 12, 18, 24 days after heading (*n* = 4 for each cultivar, *n* = 8 for each nitrogen treatment). The correlation analyses were performed based on a small number of samples in previous studies (Yang et al., 2001a; Xue et al., 2008; Xiong et al., 2015b).

## Results

### Grain Filling and Yield

**FIGURE 1:**
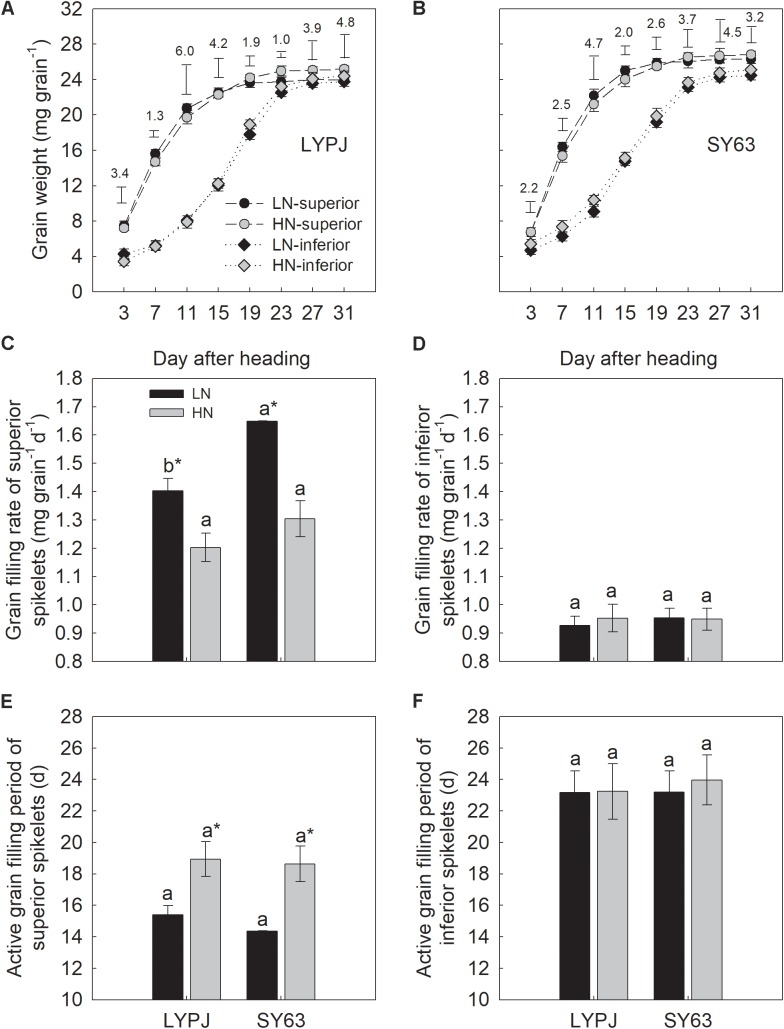
Changes in grain weight **(A,B)**, grain filling rate **(C,D)**, and active grain filling period **(E,F)** of superior and inferior spikelets in LYPJ and SY63 cultivars during the grain filling period under LN and HN treatments. Data are shown as mean ± standard error (*n* = 3). Vertical bar with number represents LSD_0.05_ for difference significance of mean among 4 combinations at an identical time point. Different letters on top of histograms indicate significance at *P* < 0.05 between the two varieties under the same nitrogen condition. ^∗^Indicates significance at *P* < 0.05 between the LN and HN treatments for the same cultivar.

Under the LN application condition, grain filling in the superior spikelets of LYPJ and SY63 was completed approximately 19 days after heading. Under the HN application condition, this process was completed around day 23. No difference was observed in the inferior spikelets of LYPJ and SY63 between the LN and HN applications (**Figures 1A,B**).

The LN treatment significantly increased the grain filling rate and shortened the active grain filling period of superior spikelets compared with the HN treatment (**Figures 1C,E**). Grain filling rate of superior spikelets increased by 16.7 and 26.4% in LYPJ and SY63, respectively (**Figure 1C**), and the active grain filling period of superior spikelets shortened by 3.5 and 4.2 days in LYPJ and SY63, respectively (**Figure 1E**). However, no significant differences were observed in active grain filling rate and grain filling period of inferior spikelets between LN and HN (**Figures 1D,F**).

The superior spikelets of SY63 exhibited a higher grain filling rate than the superior spikelets of LYPJ by 17.4 and 8.4% under the LN and HN treatments, respectively, but a significant difference was only observed under the LN treatment (**Figure 1C**). The active grain filling period of SY63 superior spikelets was 1 day less than that in LYPJ under LN, but no difference was observed under HN (**Figure 1E**). No significant differences were observed in the active grain filling period and grain filling rate of inferior spikelets of the LYPJ and SY63 cultivars (**Figures 1D,F**).

**FIGURE 2:**
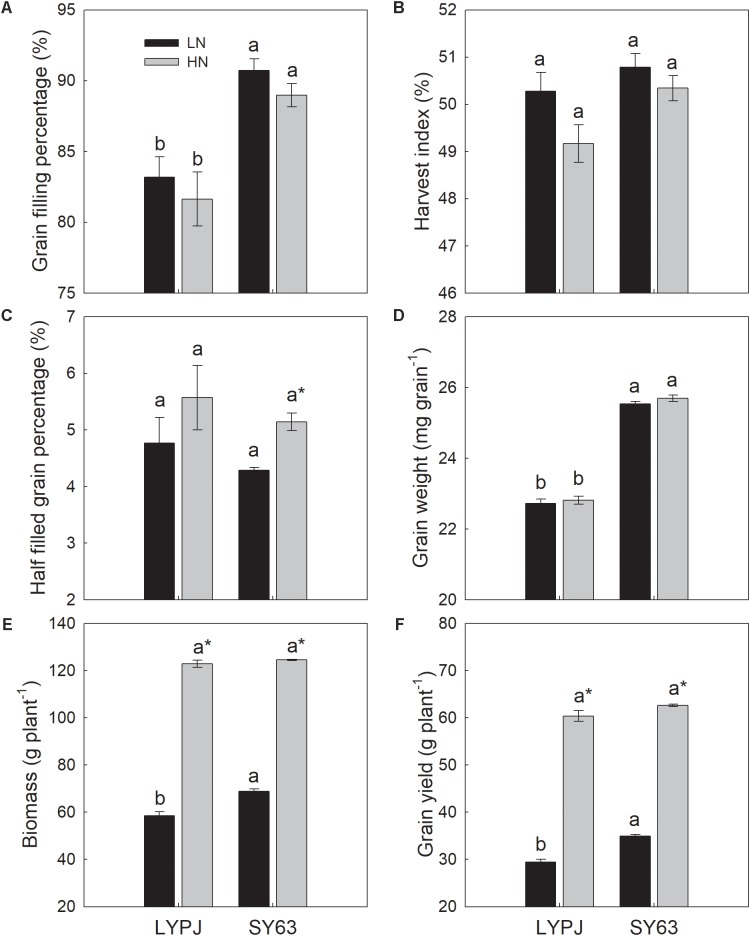
Grain filling percentage **(A)**, harvest index **(B)**, half-filled grain percentage **(C)**, grain weight **(D)**, biomass **(E)**, and grain yield **(F)** of LYPJ and SY63 cultivars under LN and HN treatments. Vertical bars represent the mean ± standard error (*n* = 3). Different letters on top of histograms indicate a significant difference at *P* < 0.05 between the two cultivars under the same nitrogen condition. ^∗^Indicates a significant difference at *P* < 0.05 between the LN and HN treatments for the same cultivar.

The LN treatment increased the grain filling percentage of LYPJ and SY63, when compared with the HN treatment, but the differences were not significant (**Figure 2A**). Low nitrogen increased the harvest index of LYPJ and SY63 (**Figure 2B**), while LN decreased the half-filled grain percentage by 14.4 and 16.6% in LYPJ and SY63, respectively; a significant difference was observed only in SY63 (**Figure 2C**). Grain weight was not influenced by nitrogen treatment (**Figure 2D**). Plant biomass and grain yield were significantly decreased under the LN treatment in both LYPJ and SY63 compared with the HN treatment (**Figures 2E,F**).

SY63 exhibited a significantly higher grain filling percentage and grain weight than LYPJ under both the LN and HN treatments (**Figures 2A,D**). Additionally, SY63 exhibited a higher harvest index than LYPJ under the LN and HN treatments, respectively (**Figure 2B**) and a lower half-filled grain percentage by 10.0 and 7.7%, respectively (**Figure 2C**); however, such differences were not significant. Higher biomass and grain yield was observed in SY63 than in LYPJ under the LN and HN treatments, but a significant difference was observed only under the LN treatment (**Figures 2E,F**).

### Changes in NSC, Starch, Soluble Sugars, and Sucrose in Stems

**FIGURE 3:**
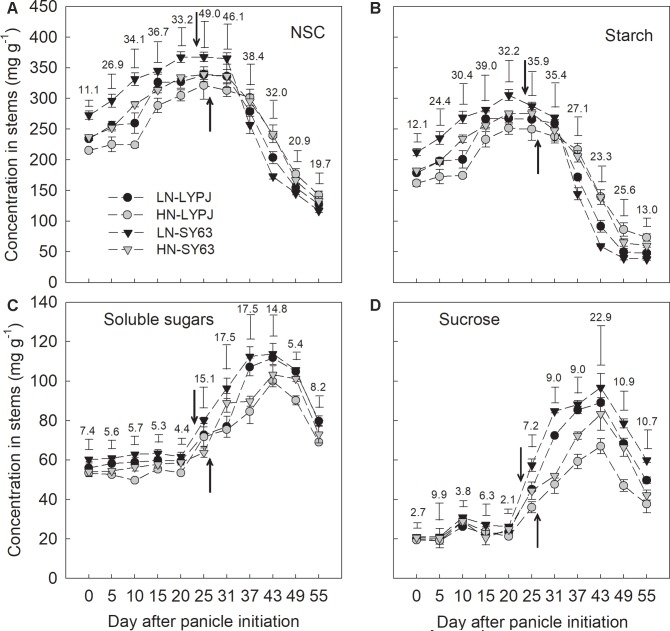
Concentrations of NSC **(A)**, starch **(B)**, soluble sugars **(C)**, and sucrose **(D)** in the stems of LYPJ and SY63 cultivars under LN and HN treatments. Vertical bar with number represents LSD_0.05_ for difference significance of mean among 4 combinations at an identical time point. The downward and upward arrows indicate heading date (HD) under LN and HN. Data are shown as mean ± standard error (*n* = 3).

Stem starch and NSC concentrations in both the SY63 and LYPJ cultivars increased between the panicle initiation and heading stages (approximately 25 days after panicle initiation) under both the LN and HN treatments, and then decreased (**Figures 3A,B**). Soluble sugars and sucrose in stems increased from the date of panicle initiation to 40 days and then decreased until maturity (**Figures 3C,D**).

Starch and NSC concentrations were higher from panicle initiation to heading under LN than under HN, and LN accelerated the disappearance of starch and NSC after heading in both LYPJ and SY63 (**Figures 3A,B**). Soluble sugars and sucrose were higher through the entire period from panicle initiation to maturity under LN than under HN (**Figures 3C,D**). The NSC and starch concentrations in SY63 were higher than in LYPJ from panicle initiation to heading and lower after heading under both the LN and HN treatments. From 6 to 24 days after heading, NSC and starch concentrations rapidly decreased in both cultivars and under both N application conditions (**Figures 3A,B**). Generally, the soluble sugar and sucrose contents of SY63 were higher than in LYPJ from panicle initiation to maturity under the LN and HN conditions (**Figures 3C,D**).

### Stem NSC Translocation

**FIGURE 4:**
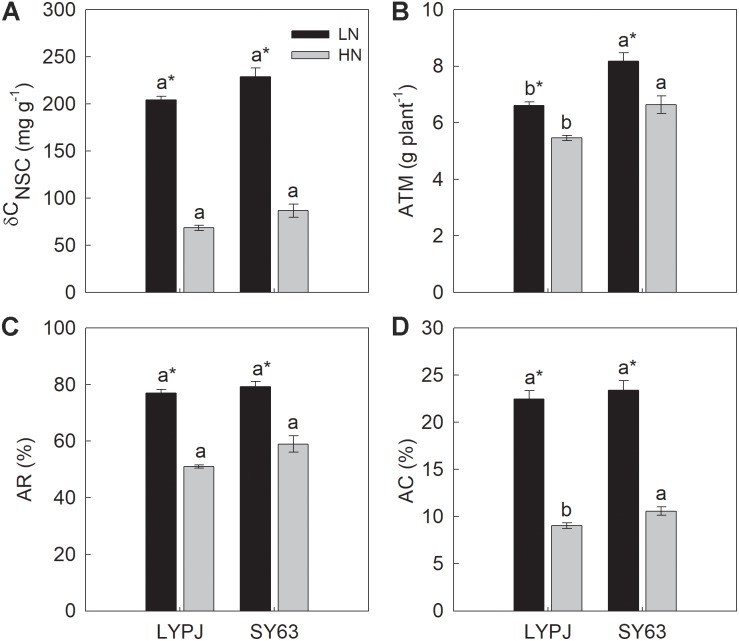
The difference of stem NSC concentrations between heading and at maturity (δC_NSC_) **(A)**, apparent transferred mass of NSC (ATM) **(B)**, apparent ratio of transferred NSC (AR) **(C)**, and apparent contribution of transferred NSC to grain yield (AC) **(D)** in LYPJ and SY63 cultivars under LN and HN treatments. Vertical bars represent mean ± standard error (*n* = 3). Different letters on top of histograms indicate significant differences at *P* < 0.05 between the two varieties under the same nitrogen condition. ^∗^Indicates significance at *P* < 0.05 between LN and HN for the same cultivar.

The four traits (δC_NSC_, ATM, AR, and AC) used to describe stem NSC translocation were significantly higher under LN than under HN in both the LYPJ and SY63 cultivars. Additionally, SY63 exhibited higher δC_NSC_, ATM, AR, and AC than LYPJ, but significant differences were observed only in ATM under both LN and HN applications and in AC under HN application (**Figures 4A–D**).

### Enzyme Activities for Starch Synthesis in Stems Before Heading

**FIGURE 5:**
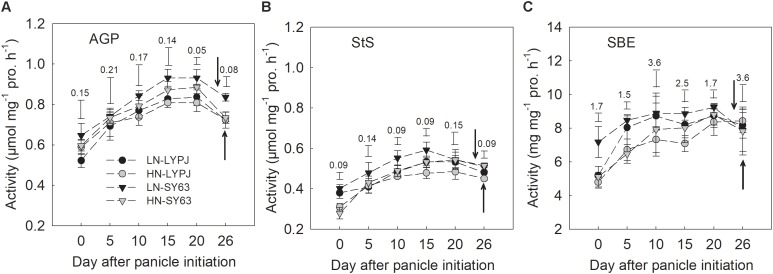
Activities of AGP **(A)**, StS **(B)**, and SBE **(C)** in the stems of LYPJ and SY63 cultivars under LN and HN treatments. Vertical bar with number represents LSD_0.05_ for difference significance of mean among 4 combinations at an identical time point. The downward and upward arrows indicate HD under LN and HN. Data are shown as mean ± standard error (*n* = 3).

The activities of AGP, StS, and SBE in stems increased from panicle initiation to heading, reaching their peak at approximately 5–10 days before heading and then decreasing. The activities of the three enzymes were higher under LN than under HN in both cultivars and higher in SY63 than in LYPJ under both N applications (**Figures 5A–C**).

### Enzyme Activities for Starch-to-Sucrose Conversion in Stems After Heading

**FIGURE 6:**
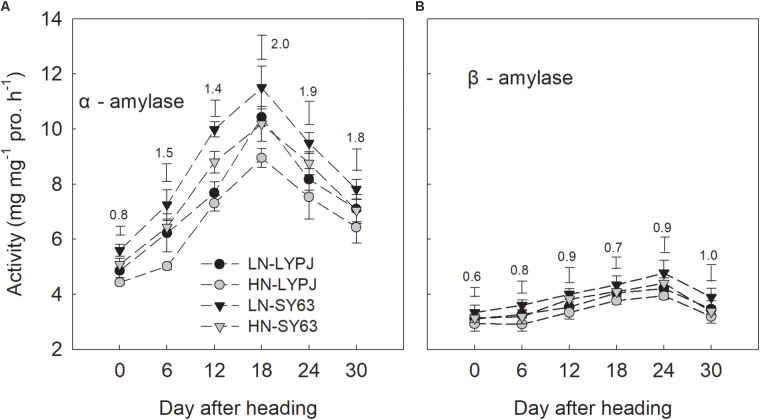
Activities of α-amylase **(A)** and β-amylase **(B)** in the stems of LYPJ and SY63 cultivars under LN and HN treatments. Data are shown as mean ± standard error (*n* = 3). Vertical bar with number represents LSD_0.05_ for difference significance of mean among 4 combinations at an identical time point.

The activities of α-amylase and β-amylase in stems increased during grain filling and were enhanced by LN. Additionally, the activity of α-amylase reached a peak 18 days after heading under both the LN and HN treatments, while the activity of β-amylase reached a peak at 24 days after heading. Overall, β-amylase activity was lower than that of α-amylase, exhibiting only small changes during grain filling. The activities of α-amylase and β-amylase were higher in SY63 than in LYPJ under both N applications (**Figures 6A,B**).

**FIGURE 7:**
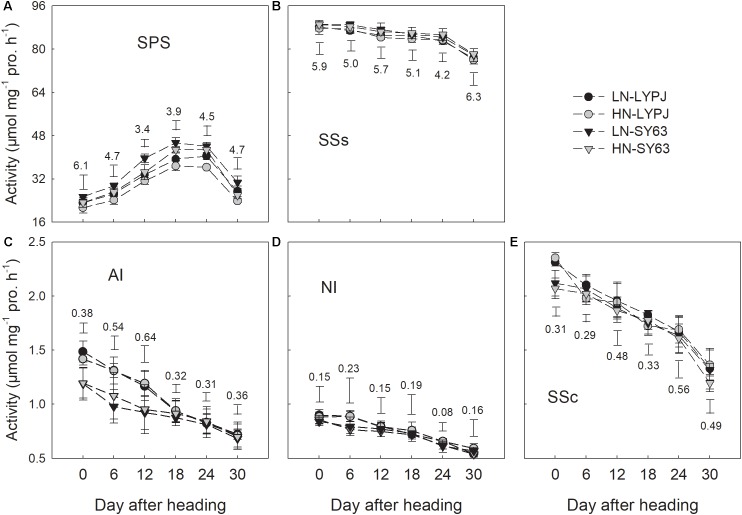
Activities of SPS **(A)**, SSs **(B)**, AI **(C)**, NI **(D)**, and SSc **(E)** in the stems of LYPJ and SY63 cultivars under LN and HN treatments. Data are shown as mean ± standard error (*n* = 3). Vertical bar with number represents LSD_0.05_ for difference significance of mean among 4 combinations at an identical time point.

The activity of SPS first increased, then maintained a relatively high level from 18 to 24 days after heading, and finally declined (**Figure 7A**). However, SSs, AI, NI, and SSc in both cultivars decreased through the grain filling stage under both the LN and HN treatments (**Figures 7B–E**). Compared with the HN treatment, the LN treatment increased SPS activity in both LYPJ and SY63 (**Figure 7A**); however, N treatment did not affect activities of SSs, AI, NI, and SSc (**Figures 7B–E**). In general, SPS activity was higher in SY63 than in LYPJ under both N application treatments (**Figure 7A**). No significant differences in the activities of SSs, AI, NI, and SSc were observed between the two cultivars, except for higher AI activity in LYPJ during the early grain filling stage and higher SSc activity in LYPJ on the day of heading (**Figures 7B–E**).

### Relationships Between Enzyme Activities and the Increase or Remobilization of Stem Starch and NSC

**FIGURE 8:**
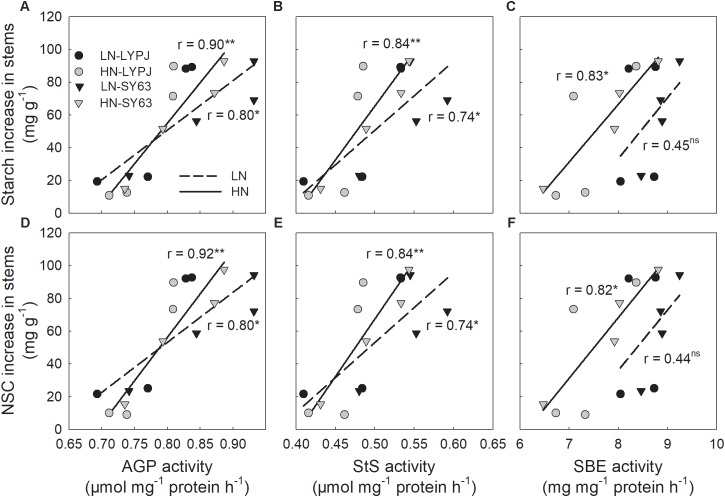
Relationships between stem starch **(A,B,C)** and NSC **(D,E,F)** accumulation and the activities of AGP, StS, and SBE during the period of rapid starch accumulation (0–20 days after panicle initiation, *n* = 4 for each cultivar, *n* = 8 for each nitrogen treatment). ns, not significant at *P* < 0.05; ^∗^ and ^∗∗^, significant at *P* < 0.05 and at *P* < 0.01, respectively.

The activities of AGP, StS, and SBE were positively correlated with increases in stem starch and NSC during panicle initiation. These correlations were significant for AGP and StS under both N application treatments, but only under the LN treatment for SBE (**Figures 8A–F**).

**FIGURE 9:**
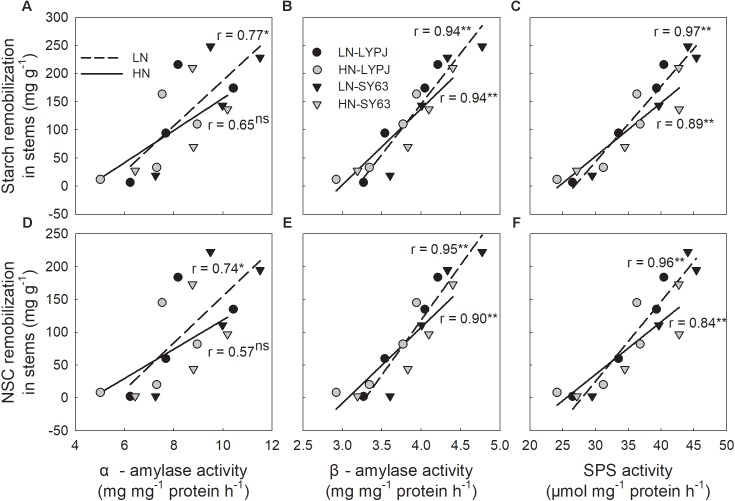
Relationships between stem starch **(A,B,C)** and NSC **(D,E,F)** remobilization and the activities of α-amylase, β-amylase, and SPS during the period of rapid starch decrease (6–24 days after heading, *n* = 4 for each cultivar, *n* = 8 for each nitrogen treatment). ns, not significant at *P* < 0.05; ^∗^ and ^∗∗^, significant at *P* < 0.05 and at *P* < 0.01, respectively.

The activities of α-amylase, β-amylase, and SPS were positively correlated with starch and NSC remobilization after heading. Most of these correlations were significant, but not the correlation between α-amylase activity and starch/NSC remobilization under HN (**Figures 9A–F**).

## Discussion

### Low Nitrogen Increased Stem NSC Accumulation by Enhancing Activities of Enzymes Involved in Starch Synthesis

Our investigations showed that LN enhanced stem starch and NSC concentrations before heading in both cultivars (**Figures 3A,B**), which was accordant to the previous studies, with the finding that stem NSC accumulation at heading decreased under HN conditions (Hirano et al., 2005; Pan et al., 2011). Under the HN condition, rice plants required more carbohydrates to build plant structures and more carbon to transform inorganic nitrogen, which could cause decreases in stem NSC at heading (Gebbing et al., 1999). In rice, starch is the main form of stem NSC before heading (Ishimaru et al., 2007); so, enzymes for starch synthesis may be involved in the process of starch accumulation. In our study, LN treatment also increased the activities of AGP, StS, and SBE enzymes before heading, which are involved in starch synthesis in stems (**Figure 5**) and varied in consistency with changes in starch and NSC concentrations (**Figures 3A,B**). Moreover, the activities of AGP, StS, and SBE enzymes positive correlated with starch and NSC concentrations (**Figure 8**), which agrees with previous results (Watanabe et al., 1997). These results suggest that enhanced activities of AGP, StS, and SBE under LN may directly lead to increases in starch and NSC concentrations in stems before heading. Hirano et al. (2005) found that low SBE activity, due to decreased expression of SBE genes, contributed to decreased starch accumulation in leaf sheaths under HN conditions. Additionally, studies have found that the activities of AGP, StS, and SBE are enhanced by water stress during the period of rapid starch accumulation in rice grains; changes in the activities of these enzymes may be an adaptive mechanism to alleviate assimilate deficiencies in times of environmental stress (Yang et al., 2003; Zhang et al., 2012). In the current study, the LN condition enhanced the activities of AGP, StS, and SBE, resulting in increased starch accumulation prior to heading. While it is clear that the LN condition creates a lower demand for carbohydrates by rice plants, due to slow growth and less transformation of inorganic nitrogen, the mechanisms underlying the effects of low N on NSC accumulation should be further investigated.

### Low Nitrogen Increased Stem NSC Translocation by Enhancing Activities of Enzymes Involved in Starch-to-Sucrose Conversion

Low nitrogen application increased stem NSC translocation during grain filling (**Figure 4**), this result is in accordance with Pan et al. (2011). In fact, starch and NSC concentrations decreased more quickly and to a greater degree under LN compared with HN (**Figures 3A,B**), indicating higher starch and NSC remobilization from stems to grains under LN. It has been reported that the remobilization of stem NSC closely correlates with senescence; plants experiencing severe senescence due to environmental stress, such as water stress or LN versus HN, exhibit a greater degree of starch and NSC remobilization than plants experiencing a lower degree of senescence (Yang et al., 2001c). Previous studies have shown that water deficits increased the translocation of NSC to grains in wheat (Yang et al., 2001b; Plaut et al., 2004) and rice (Yang et al., 2001c) and that the cause was likely due to the enhanced activities of enzymes involved in starch hydrolysis and sucrose synthesis in stems (Yang et al., 2001a). The activities of three enzymes involved in starch-to-sucrose conversion (α- and β-amylase for hydrolysis of starch, SPS for sucrose synthesis) correlated with the concentrations of soluble sugars and sucrose during the grain filling period (**Figures 3C,D, 6A,B, 7A**). The increased activities of these enzymes also appeared to enhance remobilization of stem starch and NSC (**Figures 3A,B, 4A–D, 6A,B, 7A**), which was supported by the positive correlation of the activities with the decrease in starch and NSC in stems during grain filling under the LN treatment (**Figure 9**). Although β-amylase activity was lower than α-amylase activity for the duration of the grain filling stage (**Figure 6**), β-amylase activity correlated more closely with starch and NSC remobilization (**Figures 9A,B,D,E**). β-amylase is thought to hydrolyze the oligosaccharide products of α-amylase and plays an important role in the regulation of starch hydrolysis (Gallagher et al., 1997). These results suggest that the rapid hydrolysis of starch under LN can be attributed to increased α- and β-amylase activities in stems.

Sucrose is the primary form in which photoassimilates are transported in plants (Braun et al., 2014). Rapid synthesis of sucrose, which involves the SPS and SSs enzymes, reduces concentrations of monosaccharides (Wardlaw and Willenbrink, 1994). The LN treatment enhanced SPS activity in stems during grain filling, which correlated closely with increased sucrose concentrations in stems (**Figures 3D, 7A**); this result is consistent with previous research showing that SPS is a key enzyme in sucrose synthesis (Lunn and MacRae, 2003). In addition, SPS activity correlated significantly with the remobilization of starch and NSC in stems under both N application treatments during the grain filling stage (**Figures 9C,F**). In contrast, the activity of SSs decreased gradually from heading to maturity and was not affected by N treatment; however, SSs activity was higher than SPS and remained at a high level during grain filling (**Figure 7B**), a state which is beneficial for sucrose synthesis. Overall, N treatment had no effect on the activities of enzymes involved in sucrose hydrolysis (AI, NI, and SSc) in stems; the activities of these enzymes were relatively low and decreased gradually during grain filling (**Figures 7C–E**). This suggests that AI, NI, and SSc had little effect on sucrose synthesis and the remobilization of stem starch and NSC. Based on these findings, we conclude that high activities of α-amylase, β-amylase, and SPS are the main contributors to increases in the remobilization of starch and NSC in rice stems under LN during grain filling.

It was noteworthy that superior spikelet had faster grain filling rate and shorter active grain filling period under LN (**Figure 1**). The observation was in accordance with Yang et al. (2006). However, LN had no significant effects on grain filling of inferior spikelets in our study; the similar observations were found in wheat under LN (Xu et al., 2013). Due to spikelet ontogeny and flowering time, superior spikelets often show the dominance for competing carbohydrates relatively to inferior ones, resulting in the quick and early grain filling (Yang et al., 2006). On the other hand, later-flowering inferior spikelets had the nature of hysteretic and slow grain filling; therefore, effect of nitrogen on grain filling of inferior spikelets may be diminished, especially when photosynthetic rate and available assimilates for grain filling decrease due to low N application rate.

### Genotypic Differences in Activities of Enzymes Involved in Starch Metabolization Influence NSC Accumulation and Translocation

Rice cultivars have shown large genotypic differences in their abilities to accumulate and translocate stem NSC (Samonte et al., 2001; Hirano et al., 2005; Yang and Zhang, 2010a; Pan et al., 2011). The differences in stem NSC accumulation and translocation were also observed between LYPJ and SY63 (**Figures 3A,B, 4**). Higher activities of AGP, StS, and SBE for starch synthesis in stems contributed to the higher stem starch and NSC concentrations in SY63. This result was also supported by the positive correlations between the activities of the three enzymes and the increases in starch and NSC concentrations in the two varieties in present study (**Figure 8**). Additionally, AGP and StS activities in both cultivars correlated significantly with increases in stem starch and NSC concentrations under both N application treatments (**Figure 8**); however, SY63 exhibited strong correlations between SBE activity and increases in starch (*r* = 0.97, *P* < 0.01, *n* = 4) and NSC (*r* = 0.97, *P* < 0.01, *n* = 4) under both N applications. and no such correlations were observed between SBE activity and increases in starch and NSC for the LYPJ cultivar (for starch under LN: *r* = 0.18, *P* > 0.05; for starch under HN: *r* = 0.71, *P* > 0.05; for NSC under LN: *r* = 0.18, *P* > 0.05; for NSC under LN, *r* = 0.68, *P* > 0.05). Among the starch-synthesis-related enzymes (AGP, StS, and SBE), SBE activity exhibited the highest positive correlation with starch concentration (Watanabe et al., 1997), and SBE expression correlated with starch concentration (Hirose et al., 1999). Additionally, the photosynthetic rate of LYPJ is higher than that of SY63 (Lv et al., 2017); however, LYPJ had low stem NSC and starch accumulation under both N application rates (**Figures 3A,B**). These results suggest that higher activities of AGP, StS, and SBE may contribute to higher stem starch and NSC concentrations in SY63 than LYPJ and that SBE may contribute more strongly to pre-anthesis starch and NSC accumulation in the stems of SY63 than LYPJ.

Stem NSC is an important source of assimilates used for grain filling; a high level of stem NSC at the full heading stage was found capable of maintaining enhanced sink strength and highly ripened grains (Fu et al., 2011; Morita and Nakano, 2011). Grain yield, grain weight, and grain filling percentage are closely associated with stem NSC concentration and translocation (Pan et al., 2011). SY63 had a significantly higher grain filling rate of superior spikelets and grain yield than LYPJ, especially under LN (**Figures 1C, 2A,D,F**). Our study and Yang and Zhang (2010b) suggest that better grain filling was partly attributed to higher NSC translocation. High activities of α-amylase, β-amylase, and SPS may be responsible for greater remobilization of starch and NSC in SY63 than in LYPJ, which is beneficial for grain filling after heading.

### Calculated Reductions in Nitrogen Application May Enhance Stem NSC Accumulation and Translocation

Nitrogen fertilizer application rate has crucial roles in biomass accumulation and grain yield formation. However, increasing fertilization generally result in gradual reduction in the crop yield response (Sui et al., 2013; Lassaletta et al., 2014). We noticed that the N treatments were 10 times difference between the two N rates (0.26 vs. 2.6 g N pot^-1^); however, the biomass and grain yield increased approximately twice between the N treatments (**Figures 2E,F**), suggesting that the differences in biomass and grain yields between LN and HN were not linearly proportionate with the increase of N application rates. The relationship between N application rate and grain yield in our study was in accordance with lots of previous studies in pot and field experiments. Xiong et al. (2015a) reported that grain yield was 2.0∼2.3 times higher under HN (3.5 g N pot^-1^) than LN (0.5 g N pot^-1^) in three rice varieties; Similarly, field experiment data of Chen et al. (2015) also showed that biomass and grain yield under HN (225 kg N ha^-1^) were 1.7 and 1.5 times higher than those under LN (0 kg N ha^-1^), respectively. So, the large increases in N application rates do not always result in large differences accordingly in biomass and grain yields. In fact, the responses of rice plant biomass and grain yield to N application rates may be not accumulative, and is associated with several factors, such as soil indigenous nitrogen content (soil fertility), genetic characteristics of cultivars, climate factors (radiation and temperature), cultivation managements.

The pursuit of higher rice yields often prompts the application of additional nitrogen fertilizers in many rice planting regions (Zhu and Chen, 2002), resulting in an estimated 30% overuse of nitrogen in agricultural fields in China (Ju and Gu, 2014). Besides negative consequences for the environment, nitrogen use efficiency, crop health, and grain yields (Peng et al., 2002; Peng, 2014), excess nitrogen application is not conducive to NSC accumulation and translocation, nor to high yields (He et al., 2001; Pan et al., 2011). Our study supports these observations by showing that HN resulted in lower stem NSC accumulation and translocation than LN (**Figure 4**), LN enhanced grain filling percentage and harvest index (**Figures 2A,B**), which may be attributed to the increased stem NSC translocation(**Figures 4A,B**). Although our study showed that grain yield significantly decreased under LN (**Figure 2F**), investigations by the International Rice Research Institute since 1997 have shown that appropriate reductions in nitrogen fertilizer application can simultaneously increase grain yield and nitrogen use efficiency and reach optimal productivity (Peng et al., 2002). According to our study (**Figures 3A, 4**) and reports of Pan et al. (2011) and Ju et al. (2015), LN may enhance stem NSC accumulation and remobilization, which is often beneficial for grain yield in rice. Additionally, according to the yield-nitrogen application rate relationship (Sui et al., 2013; Lassaletta et al., 2014), response of crop grain yield to N application gradually reduces with the increase of N application rate, and the contributions of N application to yield formation often become smaller at high application rates than that at low application rates. Improving nitrogen management through crop rotation in agriculturally intensive systems in China may reduce current nitrogen application rates by 30–60%, while preserving current crop yields and a healthy nitrogen balance (Ju et al., 2009). Therefore, these results suggest that it is feasible to reduce nitrogen application by using rice varieties with lower nitrogen demands, which can produce high yields even under stressful growth conditions and contribute to agricultural sustainability.

## Conclusion

In the current study, LN increased the concentrations of starch and NSC in rice stems before the plants’ heading stage. This was primarily attributed to the enhanced activities of AGP, StS, and SBE enzymes in plant stems. Reduced N application also resulted in increased activities of α-amylase, β-amylase, and SPS, which increased the remobilization of stem starch and NSC; these effects increase grain filling percentage, grain filling rate, and harvest index in rice. In terms of cultivar genotype, higher activities of AGP, StS, and SBE were responsible for higher concentrations of stem starch and NSC in the SY63 rice cultivar than in the LYPJ cultivar prior to heading; SBE played a larger role in the regulation of starch and NSC in SY63 than in LYPJ. In addition, SY63 exhibited higher activities of α-amylase, β-amylase, and SPS, which increased stem starch and NSC translocation; these effects increase grain filling percentage, grain filling rate, grain weight, and grain yield in SY63. Our results suggest that calculated reductions in nitrogen application may improve grain yields via enhanced stem NSC accumulation and translocation, thereby reducing the costs and increasing the sustainability of rice production.

## Author Contributions

GL and KC conceived the research, designed the experiments, analyzed the data, and wrote the manuscript. GL carried out pot experiments. QH and YS assisted in both sampling and physiological determinations in the laboratory. SP, JH, and LN gave valuable suggestions during the whole pot and laboratory experiments.

## Conflict of Interest Statement

The authors declare that the research was conducted in the absence of any commercial or financial relationships that could be construed as a potential conflict of interest.

## ABBREVIATIONS

AC: apparent contribution of transferred NSC to grain yield
AGP: adenosine diphosphate-glucose pyrophosphorylase
AI: acid invertase
AR: apparent transferred ratio of NSC from stems to grains
ATM: apparent transferred mass from stems to grains
HN: high nitrogen application
LN: low nitrogen application
NI: neutral invertase
NSC: non-structural carbohydrates
SBE: starch branching enzyme
SPS: sucrose phosphate synthase
SSc: sucrose synthase in the cleavage direction
SSs: sucrose synthase in the synthetic direction
StS: starch synthase
